# Brevican and Neurocan Cleavage Products in the Cerebrospinal Fluid - Differential Occurrence in ALS, Epilepsy and Small Vessel Disease

**DOI:** 10.3389/fncel.2022.838432

**Published:** 2022-04-11

**Authors:** Wilhelm Hußler, Lukas Höhn, Christopher Stolz, Stefan Vielhaber, Cornelia Garz, Friedhelm C. Schmitt, Eckart D. Gundelfinger, Stefanie Schreiber, Constanze I. Seidenbecher

**Affiliations:** ^1^Leibniz Institute for Neurobiology (LIN), Magdeburg, Germany; ^2^Department of Neurology, Otto-von-Guericke University Magdeburg, Magdeburg, Germany; ^3^Center for Behavioral Brain Sciences (CBBS), Magdeburg, Germany; ^4^Institute for Pharmacology and Toxicology, Otto-von-Guericke-University Magdeburg, Magdeburg, Germany; ^5^German Center for Neurodegenerative Diseases (DZNE), Magdeburg, Germany

**Keywords:** cerebral small vessel disease (CSVD), amyotrophic lateral sclerosis (ALS), extracellular matrix (ECM), biomarker, CNS, serum

## Abstract

The neural extracellular matrix (ECM) composition shapes the neuronal microenvironment and undergoes substantial changes upon development and aging, but also due to cerebral pathologies. In search for potential biomarkers, cerebrospinal fluid (CSF) and serum concentrations of brain ECM molecules have been determined recently to assess ECM changes during neurological conditions including Alzheimer’s disease or vascular dementia. Here, we measured the levels of two signature proteoglycans of brain ECM, neurocan and brevican, in the CSF and serum of 96 neurological patients currently understudied regarding ECM alterations: 16 cases with amyotrophic lateral sclerosis (ALS), 26 epilepsy cases, 23 cerebral small vessel disease (CSVD) patients and 31 controls. Analysis of total brevican and neurocan was performed *via* sandwich Enzyme-linked immunosorbent assays (ELISAs). Major brevican and neurocan cleavage products were measured in the CSF using semiquantitative immunoblotting. Total brevican and neurocan concentrations in serum and CSF did not differ between groups. The 60 kDa brevican fragment resulting from cleavage by the protease ADAMTS-4 was also found unchanged among groups. The presumably intracellularly generated 150 kDa C-terminal neurocan fragment, however, was significantly increased in ALS as compared to all other groups. This group also shows the highest correlation between cleaved and total neurocan in the CSF. Brevican and neurocan levels strongly correlated with each other across all groups, arguing for a joint but yet unknown transport mechanism from the brain parenchyma into CSF. Conclusively our findings suggest an ALS-specific pattern of brain ECM remodeling and may thus contribute to new diagnostic approaches for this disorder.

## Introduction

In the healthy adult human brain approximately 20-22% of the neural parenchyma represent extracellular space (Vargova et al., 2011; Nicholson and Hrabìtová, 2017). This space is not occupied by neural cells but filled with interstitial fluid and with components of the extracellular matrix (ECM) produced by neurons and glial cells. Typical components of the brain ECM are hyaluronic acid, heparan sulfate and chondroitin sulfate proteoglycans (CSPGs), link proteins and glycoproteins like tenascins. Among the CSPGs the lectican family including aggrecan, brevican, neurocan and versican is of utmost importance (Frischknecht et al., 2014). After secretion of ECM building blocks into the extracellular space, these molecules form structurally and functionally diverse three-dimensional meshworks surrounding and insulating neuronal somata and neurites, including synapses or – as perinodal ECM – the nodes of Ranvier and axon initial segments. Thus, they generate defined extracellular compartments. The most peculiar forms of neural ECM are the perineuronal nets (PNN) largely found on parvalbumin-positive inhibitory neurons, but there is also a more diffuse form of ECM filling the entire extracellular space and a laminin/collagen-based perivascular ECM associated with brain microvessels (for review see Ulbrich et al. (2021)).

ECM structures create microcompartments for stem cell migration, diffusion processes, for the presentation of trophic factors to their cognate receptors and for synaptic and volume transmission (summarized in Dityatev et al. (2010)). As demonstrated by a plethora of studies, physiological plasticity processes in the brain like ocular dominance plasticity, remote fear memory or cognitive flexibility are affected by ECM-disintegrating enzymatic treatments (Pizzorusso et al., 2002; Happel et al., 2014; Thompson et al., 2018), and vice versa synaptic activity can actively induce controlled ECM proteolysis, thus locally shaping ECM subcompartments (Mitlöhner et al., 2020).

**FIGURE 1:**
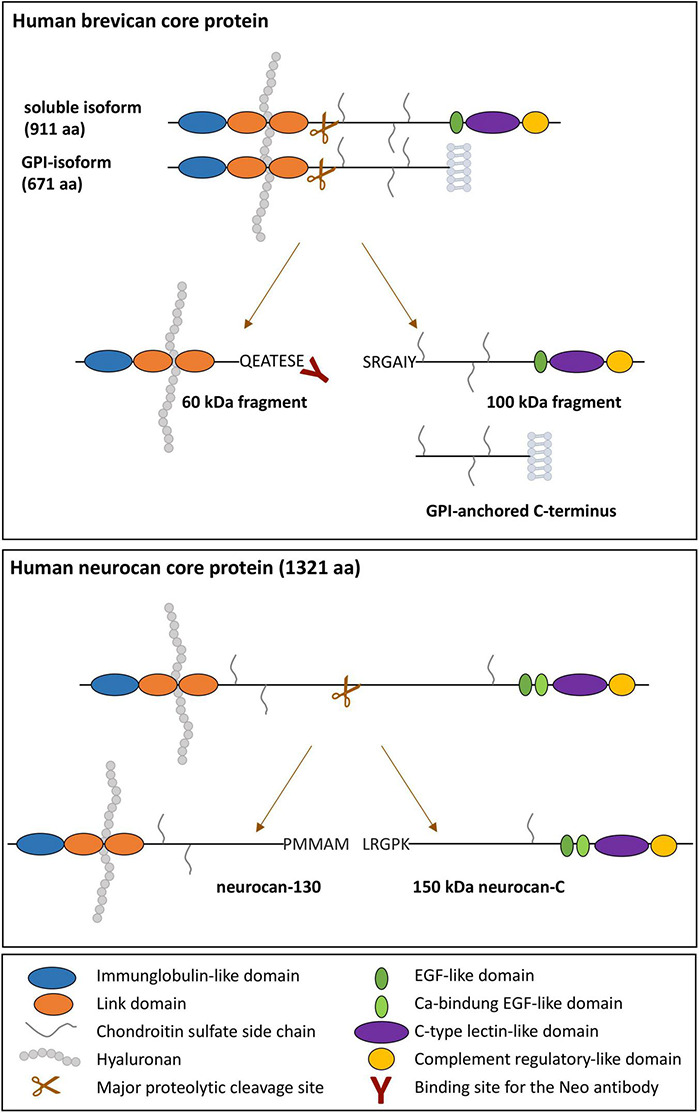
Schematic illustration of the domain structure of human soluble and GPI-anchored brevican and neurocan and their major proteolytic fragments. Domains are color-coded. The hyaluronan-binding regions and the binding site of the neoepitope-specific brevican antibody are indicated.

Indeed, hyaluronan-binding proteoglycans neurocan and brevican are substrates for controlled proteolytic cleavage by matrix metalloproteases (MMPs) or ‘A Disintegrin and Metalloproteinase with Thrombospondin motifs’ (ADAMTS) enzymes. Like all lecticans, they have in common a dumbbell-like three-dimensional protein structure with hyaluronan-binding domains localized in the N-terminal globular parts and cell-surface- or glycoprotein-interacting domains in the C-terminus, such that proteolytic events within the central rod-like domain separate proteoglycan parts and loosen the ECM integrity (Rauch et al., 2001; Frischknecht and Seidenbecher, 2012; Figure 1). The major neurocan fragments of 150 and 130 kDa are thought to be generated intracellularly and independent of ADAMTS activity (Asher et al., 2000). In contrast to neurocan, brevican occurs also as a glycosylphosphatidylinositol (GPI)-anchored minor isoform (Seidenbecher et al., 1995), which constitutes a substrate for ADAMTS-4 cleavage as well, giving rise to the same 60 kDa N-terminal fragment as the soluble isoform. Thus, proteolytic cleavage separates ECM-binding from cell surface-binding entities, leading to disintegration of the cell-ECM-meshwork. This is a dynamic and tightly controlled process to maintain the extracellular homeostasis of the brain.

Under aging and pathophysiological conditions the integrity and composition of the brain ECM undergo extensive changes, leading to a disturbed equilibrium of ECM production and deposition on one hand and ECM cleavage and clearance on the other hand (Ulbrich et al., 2021). In human epilepsy as well as in rodent epilepsy models manifold ECM remodeling phenotypes have been reported in the brain tissue, with a particular focus on PNN disruption due to increased proteolysis, which may contribute to epileptogenesis (summarized in Dityatev et al. (2010), Chaunsali et al. (2021)). In histochemical analyses of postsurgical specimen from focal cortical dysplasia patients, known for a longstanding epilepsy with a high seizure burden, a complete reorganization of the ECM was reported with reduced brevican expression and disturbance of extracellular space diffusion parameters (Zamecnik et al., 2012).

For amyotrophic lateral sclerosis (ALS), an adult-onset neurodegeneration of motor neurons, the picture is less clear, but studies in animal models revealed that in the surrounding of affected motor neurons a non-permissive microenvironment for regeneration is formed. In the lesion area in the adult spinal cord of a rat ALS model this microenvironment was shown to contain accumulations of chondroitin sulphate proteoglycans neurocan and versican (Mizuno et al., 2008). In the superoxide dismutase 1 (SOD1) model of ALS also a dramatic reduction of neuron-protecting PNNs (Forostyak et al., 2014) and a shift in CSPG receptor expression from neurons to glial cells was shown (Shijo et al., 2018). In the serum and CSF of ALS patients MMPs 2 and 9 and their inhibitors were found to be dysregulated (Niebroj-Dobosz et al., 2010) and a quantitative proteomic study of ALS patients’ CSF samples revealed significant disturbance in ECM regulatory protein networks (Collins et al., 2015).

Sporadic and familial Cerebral Small Vessel Diseases (CSVDs), a spectrum disorder affecting blood-brain barrier and small vessel wall integrity, are also hypothesized to be linked to dysregulated ECM and its proteolytic fragmentation (Vilar-Bergua et al., 2016; Benveniste and Nedergaard, 2021). CSVDs are considered a major risk factor for vascular cognitive impairment (Schreiber et al., 2020). Dysregulated ECM in the brain tissue of human CSVDs comprises heparan sulfate proteoglycans (van Horssen et al., 2001), Tissue Inhibitor of Matrix Metalloproteases TIMP1 or TIMP3 and the ECM receptor CD44 (Monet-Leprêtre et al., 2013; Manousopoulou et al., 2017; Grand Moursel et al., 2018), overall arguing for a dysbalance of controlled proteolytic ECM dynamics along the CSVD spectrum.

Just recently, perineuronal ECM components, like brevican and neurocan, have been detected in the serum and CSF of humans, providing the opportunity to study their concentration *in vivo* and establish new biomarkers as a proxy for neural ECM reorganization. Thus far, serum and CSF biomarker studies have focused on traumatic brain injury (TBI), on vascular and neurodegenerative dementia and have shown lower CSF brevican, comprising its N-terminal peptides, and neurocan levels as well as decreased total brevican, but an increase of its C-terminal fragments in the patients’ serum (Minta et al., 2019a,2021a,2021b; Jonesco et al., 2020).

Based on the fact, that in CSVDs, epilepsy, and ALS, three typical neurological disorders in the Western population, ECM dysregulations have been reported, we focused on these disorders in our study. We compared the CSF and serum concentrations of brevican, neurocan and their major cleavage products between the disease groups and a control group, and searched for correlations with further demographic and serological factors as confounders. Our main objective was to detect group differences, i.e., disease-specific ECM signatures in the CSF, focusing on cleaved brevican and neurocan, as a proxy for dysregulation in neural ECM turnover.

## Materials and Methods

### Patient Cohort, Neurological Assessment

The study included a cohort of 96 patients comprising 16 with ALS, 26 with focal epilepsy, 23 with CSVD and 31 controls (Table 1). Note that subgroups differed slightly in n-numbers due to a few missing samples. Exact n numbers are given in all figure captions.

**TABLE 1:** Demographic data of the patient cohorts.

Patients		CSF samples			Serum samples		
N	Group	n	Male/Female (%)	Mean Age ± SD	n	Male/Female (%)	Mean Age ± SD
**31**	**Controls**	**26**	46.2/53.8	60.5 ± 15.5	**26**	42.3/57.7	62.3 ± 13.6
**16**	**Amyotrophic Lateral Sclerosis (ALS)**	**16**	43.8/56.2	64.4 ± 13.2	**10**	40.0/60.0	63.0 ± 14.9
**26**	**Epilepsy**	**25**	44.0/56.0	54.7 ± 18.6	**26**	46.2/53.8	54.3 ± 18.4
**23**	**Cerebral Small Vessel Disease (CSVD)**	**22**	68.2/31.8	72.6 ± 9.4	**22**	72.7/27.3	72.8 ± 9.5
11	Cerebral Amyloid Angiopathy (CAA)	11	54.5/45.5	74.0 ± 4.9	10	60.0/40.0	73.9 ± 5.2
12	Hypertensive Arteriopathy (HA)	11	81.8/18.2	71.2 ± 12.6	12	83.3/16.7	71.8 ± 12.2
**96**	**Total cases**	**89**	**50/50**	**62.6 ± 16.1**	**84**	**51.2/48.8**	**62.7 ± 15.9**

*Bolded numbers indicates sum up to the total. CAA and HA are subcategories of CSVD.*

Patients were recruited from the Department of Neurology at the Otto-von-Guericke University Magdeburg between 02/2012 and 03/2020. ALS diagnosis was based on the revised El Escorial criteria (Brooks et al., 2000; Carvalho and Swash, 2009). According to these, 3 patients suffered from possible, 8 from probable and 5 from definite ALS within the ALS cohort. Epilepsy patients were diagnosed with focal epilepsy according to the International League Against Epilepsy (ILAE) (Scheffer et al., 2017). The CSVD cohort comprised 11 cases with probable CAA diagnosed according to the modified Boston criteria; four of them fulfilled a concomitant clinical diagnosis of Alzheimer’s disease (AD) according to the NINDCS/ADRDA criteria (Linn et al., 2010; McKhann et al., 1984). Another 12 CSVD patients had HA and diagnosis was based on the existence of deep and mixed, i.e., deep and lobar hemorrhages, detected on T2*-weighted magnetic resonance imaging (MRI) sequences (Pasi et al., 2018; Scheumann et al., 2020).

A hospital-based cohort of neurologic patients, comprising cases with non-specific complaints who underwent lumbar puncture in terms of a diagnostic workup to rule out any neurologic condition served as controls. None of those disease controls suffered from any neurological disorder, especially not from ALS, epilepsy or seizures, CAA or AD, and HA.

### Sample Taking and Biomarker Measurements

In all patients and controls lumbar puncture (LP) and venous puncture (VP) have been conducted for diagnostic workup. For lumbar puncture patients were seated and 9 ml CSF were taken. In all samples, relevant CSF blood contamination was excluded visually/macroscopically and through microscopy of non-centrifugated CSF aliquots. After centrifugation, the pellet was separated to avoid the presence of cells in the final analysis samples. Within 20 min of LP and VP, CSF and serum samples were centrifuged at 4°C, aliquoted and stored at −80°C until brevican and neurocan analysis (see below). In ALS and CSVD, CSF markers of neuroaxonal damage and neurodegeneration were determined immediately after sample collection; they were not available in the epilepsy group. Neurofilament light chain (NF-L) was measured with commercially available ELISA (NF-light^®^ ELISA, IBL International GmbH, Hamburg, Germany) and total Tau (tTau) was determined either through ELISA (Innotest hTauAg, Innogenetics, Ghent, Belgium (until 12/2019)) or with an automated immunoassay (LUMIPULSE^®^ G600 II, Fujirebio lnc., Japan (from 01/2020 on)), following the manufacturer’s instructions (see also Schreiber et al. (2018a,b)). CSF Abeta 1-40 was assessed with the Innotest β-Amyloid(1-40) ELISA (until 12/2019) and after 1/2020 also with the automated immunoassay. CSF and serum albumin as well as Immunoglobulin G were determined by rate nephelometry (Nephelometer Immage 800, Beckman Coulter, Fullerton, CA, United States). The CSF albumin/serum albumin ratio (Q_*alb*_ x 10^–3^) was calculated to assess blood-brain barrier (BBB) integrity, and the IgG-Index (Q_*alb*_/Q_*IgG*_) was calculated to assess intrathecal immunoglobulin synthesis as a predictor for neuroinflammatory processes.

### Ethics and Informed Consent

The study was approved by the Ethics Committee of the Otto-von-Guericke-University Magdeburg (Sign 07/17). Patients donating biological material gave written informed consent in accordance with the Declaration of Helsinki. Data and material were handled in a coded fashion guaranteeing patient anonymity.

### Enzyme-Linked Immunosorbent Assay Protocols

For the quantitative measurement of total brevican and neurocan we used commercial anti-human brevican und anti-human neurocan ELISA kits, respectively (RayBiotech Norcross, GA, United States) (ELH-BCAN-1 und ELH-NCAN-1). Serum samples were diluted 1:2 for brevican ELISAs and used undiluted for neurocan ELISAs. The dilution factor for CSF was 1:200 to 1:300 for anti-brevican ELISAs (to optimize the dilution for the detection range of the ELISA batches) and 1:10 for anti-neurocan ELISAs. ELISA measurements were essentially conducted according to the manufacturer’s instructions. In brief, pre-diluted CSF and serum samples were incubated in 96-well strip microplates pre-coated with monoclonal mouse anti-brevican (immunogen: aa 23-911) or polyclonal sheep anti-neurocan (immunogen: aa 23-1321). Samples were analyzed as triplicates or duplicates.

To compensate for sensitivity differences in ELISA batches the calibrator concentration of the recombinant brevican or neurocan protein for the standard curve was adjusted according to OD values obtained (highest standard concentrations were set to 3 ng/ml in brevican ELISAs and 25 ng/ml in neurocan ELISAs; standard dilution series: 6 × 1:2 dilution steps).

After 2.5 h incubation, 1:80 diluted biotinylated antibody against the target protein was added and incubated for 1 h, followed by a 45 min incubation with HRP-streptavidin (1:200 for brevican and 1:500 for neurocan) and a 30 min incubation with 3,3′,5,5′-tetramethylbenzidine in the dark. The reaction was stopped by adding 0.2 M H_2_SO_4_. OD values were measured in a VersaMax™ Tunable Microplate Reader (Molecular Devices, San Jose, United States) at 450 nm and at 650 nm for unspecific background subtraction. According to the manufacturer’s information intra- and inter-assay variability is below 10 and 12%, respectively.

### Antibodies

Following primary antibodies were used: rabbit anti-brevican “neo” (Rb399) (custom-made; rat neo-epitope QEAVESE; see Valenzuela et al., 2014; 1:500), polyclonal sheep anti-human/rat brevican (AF4009, R&D Systems, Minneapolis, United States, 1:1000), polyclonal sheep anti-rat/mouse neurocan (AF5800, R&D Systems, Minneapolis, United States, 1:1000), and the antibodies from the ELISA kits (see above).

To prove if the neo-antibody, which was originally produced against the neo-epitope of the rat N-terminal brevican fragment, is also detecting the human brevican fragment neo-epitope EATESE we performed a competition assay with pre-incubation of the primary antibody with a 100-fold molar excess of the peptide Biotin-Ahx-CGQEAVESE containing the immunogen for 1 h at RT. As shown in Supplementary Figure 1, the 60 kDa band detected by this antibody in human CSF samples is clearly diminished in the peptide competition condition, demonstrating its specificity.

As secondary antibodies we used peroxidase-conjugated AffiniPure donkey anti-rabbit IgG (H + L) and peroxidase-conjugated AffiniPure donkey anti-sheep IgG (H + L) (Jackson ImmunoResearch, Cambridgeshire, United Kingdom, 1:2000).

### Immunoblotting

For semiquantitative assessment of brevican and neurocan fragments undiluted CSF samples were incubated with chondroitinase ABC (Sigma-Aldrich, St. Louis, United States) at 1U/mg at 37°C for 30 min to digest chondroitin sulfate side chains and afterward solubilized in 5 × SDS loading buffer (250 mM Tris/HCl, pH 8, 50% glycerol, 10% SDS, 0.25% bromophenol blue, 0.5 M DTT) and boiled at 95°C for 10 min. Samples were separated on 2,2,2-trichloroethanol (TCE)-containing stain-free 5–20% Tris-glycine SDS polyacrylamide gels under reducing conditions. Before Western blotting proteins were activated under UV light for 5 min. Protein transfer onto PVDF membranes (Merck Millipore, Burlington, MA, United States) was performed according to standard protocols. To control for proper protein transfer UV images of the membranes were captured. After blocking for 1 h at RT in 5% horse serum (Sigma-Aldrich, St. Louis, United States) in TBS-T (150 mM sodium chloride, 50 mM Tris, 0.1% (v/v) Tween20, pH 7.6) membranes were incubated in primary antibodies overnight at 4°C. After washing three times with TBS-T for 10 min, secondary antibodies were added and incubated for 60 min at RT. After washing membranes again three times with TBS-T for 10 min, immunodetection was performed using an ECL Chemocam Imager (INTAS Science Imaging Instruments GmbH, Göttingen, Germany). To improve semiquantitative comparability of optical density data from immunoblots we used a standard sample loaded on all gels as a reference to calibrate individual blot data and compensate for gel differences. Quantification of band intensities was done using NHI ImageJ software version 1.52a (US National Institutes of Health, Bethesda, MD, United States).

### Statistical Analysis of Data

For the analysis of brevican and neurocan levels non-parametric testing was used due to the sample characteristics, particularly the relatively small sample sizes of the ALS and CSVD groups. Because the overall groups were not matched for age and sex, a rank analysis of covariance was performed on brevican and neurocan levels following the protocol of Quade (1967). To this end, the data were transformed into rank coefficients and sex and age were partialled out of the respective dependent variable (brevican and neurocan levels) using a multiple regression. The resulting residuals of the dependent variable were z-standardized and entered a one-way ANOVA including the factor group (control, ALS, epilepsy, and CSVD). Given a significant group effect, *post hoc* comparisons were conducted using Tukey’s multiple comparison tests (Tukey’s HSD). For statistical comparisons within the CSVD (HA versus CAA) group, t-tests were calculated on age- and sex-corrected brevican and neurocan levels. For analyses of data uncorrected for sex and age, please see Supplementary Tables 1, 2. For assessment of correlations on the uncorrected data, Spearman’s rank correlation (r_ρ_) was used and p values were reported with Bonferroni correction for multiple testing. The level of significance was defined as *p* ≤ 0.05 and all tests were conducted two-tailed. Results were reported with Cohen’s d and partial eta squared (η_*p*_^2^) as effect sizes. Statistical analysis was done using GraphPad Prism version 9 (GraphPad Software, Inc., San Diego. CA, United States) and SPSS Version 28 (IBM Corp., Armonk, NY, United States).

## Results

### Brevican and Neurocan Levels in the Cerebrospinal Fluid and Serum of Patient Groups

**FIGURE 2:**
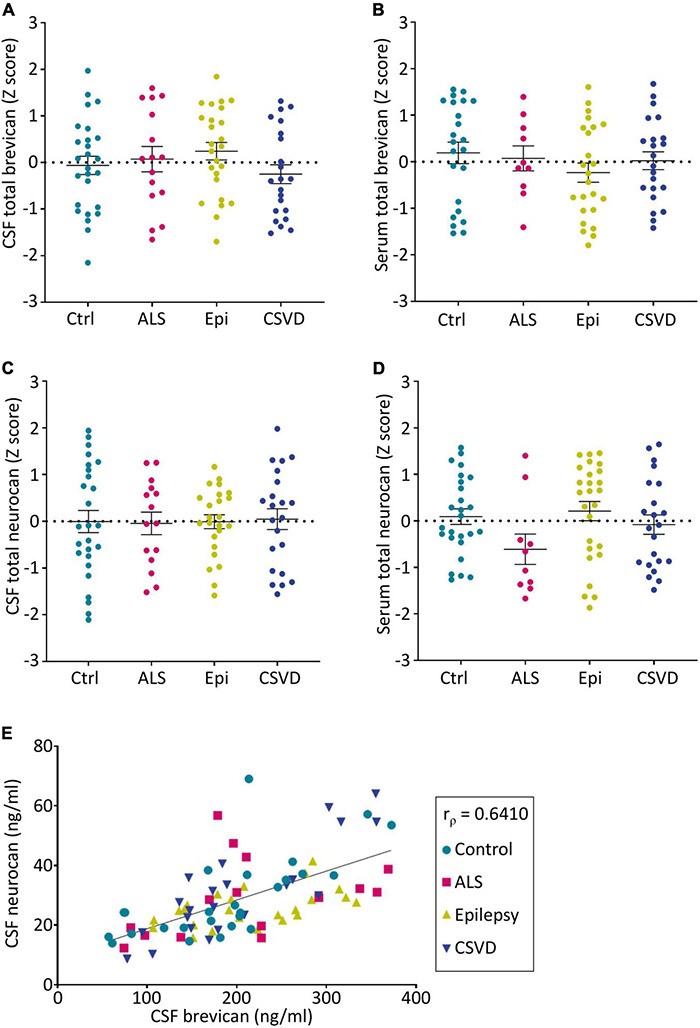
Group-wise comparison of z-scores from age- and sex-corrected total levels of brevican **(A,B)** and neurocan **(C,D)** as measured *via* ELISAs in CSF **(A,C)** and serum **(B,D)** samples (CSF BCAN: Ctr. *n* = 26, ALS *n* = 16, Epi *n* = 25, CSVD *n* = 22; CSF NCAN: Ctr. *n* = 26, ALS *n* = 15, Epi *n* = 24, CSVD *n* = 22; Serum BCAN: Ctr. *n* = 23, ALS *n* = 10, Epi *n* = 24, CSVD *n* = 22; Serum NCAN: Ctr. *n* = 26, ALS *n* = 10, Epi *n* = 26, CSVD *n* = 22). No statistical significances were detected between groups. **(E)** The concentrations of total brevican and total neurocan in the CSF correlate with each other (r_ρ_ = 0.6211, *p* < 0.0001). CSF, cerebrospinal fluid; Ctrl, control; ALS, amyotrophic lateral sclerosis; Epi, epilepsy; CSVD, cerebral small vessel disease.

The concentrations of total neurocan and total brevican were quantitatively assessed in patients’ body fluids with commercially available ELISAs. For all patient groups the mean values ± SD and the median values of raw data are given in Table 2. These levels are in the same concentration range as documented in previous reports (Minta et al., 2019a; Jonesco et al., 2020). It has to be noted, though, that serum concentrations of neurocan were at the lower detection limit of the assay in many cases. The rank analysis of covariance including the factor group (control, ALS, epilepsy, & CSVD) did not reach statistically significance on both, brevican, *F*(3,85) = 1.04, *p* = 0.38, η_*p*_^2^ = 0.04, and neurocan total CSF concentration levels, *F*(3,83) = 0.03, *p* = 0.99, η_*p*_^2^ < 0.01; see Figures 2A-D. The effect of group was also not significant for brevican total serum levels, *F*(3,75) = 0.75, *p* = 0.53, η_*p*_^2^ = 0.03, and for neurocan total serum levels, *F*(3,80) = 1.84, *p* = 0.15, η_*p*_^2^ = 0.07. No significant differences were measured within the CSVD subgroups (CAA versus HA) for brevican and neurocan total CSF concentration levels (all *t*s ≤ 1.40, ps ≥ 0.18) and total serum concentration levels (all *t*s ≤ 0.86, ps ≥ 0.40).

**TABLE 2:** CSF and serum concentrations of total brevican and neurocan in all groups.

	CSF samples						Serum samples					
Group	Neurocan			Brevican			Neurocan			Brevican		
	n	Mean ± SD (ng/ml)	Median	n	Mean ± SD (ng/ml)	Median	n	Mean ± SD (ng/ml)	Median	n	Mean ± SD (ng/ml)	Median
**Controls**	**26**	29.26 ± 14.12	24.25	**26**	190.4 ± 83.98	196.2	**26**	0.59 ± 0.079	0.39	**23**	1.766 ± 0.693	1.712
**Amyotrophic Lateral Sclerosis (ALS)**	**15**	29.16 ± 13.06	29.27	**16**	208.5 ± 91.88	198.2	**10**	0.7 ± 1.63	0.14	**10**	1.665 ± 0.568	1.557
**Epilepsy**	**24**	25.83 ± 6.18	25.10	**25**	208.9 ± 73.33	202.8	**26**	0.92 ± 1.61	0.46	**24**	1.55 ± 0.76	1.377
**Cerebral Small Vessel Disease (CSVD)**	**22**	31.20 ± 15.51	28.79	**22**	200.9 ± 81.98	176.8	**22**	0.44 ± 0.53	0.29	**22**	2.05 ± 2.365	1.644
Cerebral Amyloid Angiopathy (CAA)	11	28.04 ± 8.234	29.90	11	178.8 ± 54.54	173.1	10	0.49 ± 0.73	0.19	10	2.559 ± 3.501	1.553
Hypertensive Arteriopathy (HA)	11	34.37 ± 20.37	26.00	11	223.0 ± 100.3	208.4	12	0.40 ± 0.31	0.33	12	1.626 ± 0.47	1.644
**Total cases**	**87**	**28.79 ± 12.6**	**25.45**	**89**	**201.5 ± 81.08**	**193.7**	**84**	**0.67 ± 1.17**	**0.33**	**79**	**1.77 ± 1.37**	**1.636**

*Bolded numbers indicates sum up to the total. CAA and HA are subcategories of CSVD.*

**FIGURE 3:**
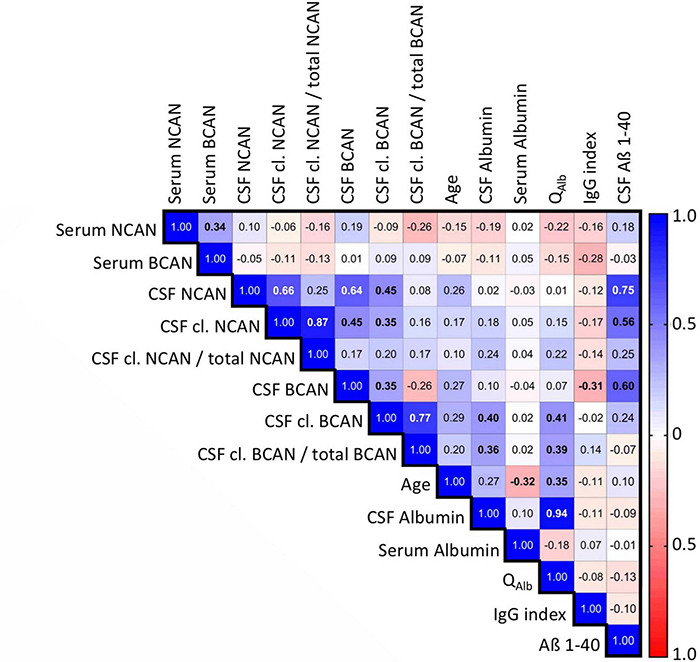
Spearman’s correlation matrix of the concentrations of ECM proteoglycans brevican and neurocan in the CSF and serum of all patients including controls with demographic and serological factors measured during diagnostic work up. Values show the Spearman rank results (significant correlations are indicated in bold). R values are color-coded with blue colors showing positive correlations, red showing negative correlations, and white showing insignificant correlations. BCAN, brevican; NCAN, neurocan; cl., cleaved; Q_*Alb*_, albumin quotient; IgG index, Immunoglobulin G index.

Interestingly, the levels of neurocan and brevican in the CSF showed a significant positive correlation (r_ρ_ = 0.641, *p* < 0.001) which was observable in all sample groups (Figure 2E). There was also a significant correlation between the serum total neurocan and total brevican concentrations (r_ρ_ = 0.34, *p* < 0.05; not shown as a separate plot, but indicated in Figure 3).

### Levels of Brevican and Neurocan Fragments in the Cerebrospinal Fluid

To assess the relative abundance of major proteoglycan fragments, i.e., the 60 kDa N-terminal brevican fragment derived from ADAMTS-4 cleavage and the 150 kDa neurocan-C fragment produced independently from ADAMTS activity, we used immunoblots with chondroitinase ABC-digested CSF samples and a reference sample for calibration. Since brevican and neurocan fragment concentrations in the serum samples were too low to be reliably detected on immunoblots, we focused on CSF samples.

#### Cleaved Brevican in the Cerebrospinal Fluid

**FIGURE 4:**
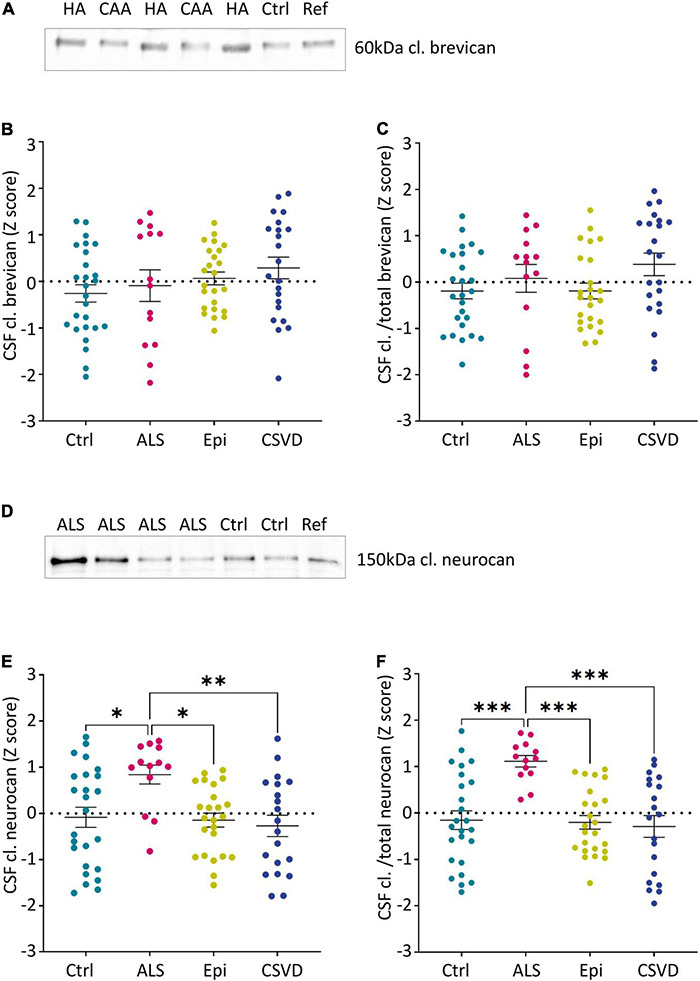
Group-wise comparison of z-scores from age- and sex-corrected CSF levels of the 60 kDa brevican fragment **(A–C)** and the 150 kDa neurocan-C fragment **(D–F)** as measured *via* immunoblots (normalized to controls). Cutouts of example western blots of cleaved brevican **(A)** and neurocan **(D)** immunoreactivity in individual CSF samples and a reference sample loaded on all gels for calibration. **(C)** and **(F)** show the ratios of cleaved brevican **(B)** and cleaved neurocan **(E)** to the total immunoreactivity determined for these proteoglycans (CSF cl.BCAN and cl./total BCAN: Ctr. *n* = 26, ALS *n* = 14, Epi *n* = 24, CSVD *n* = 22; CSF cl. NCAN and CSF cl./total NCAN: Ctr. *n* = 25, ALS *n* = 13, Epi *n* = 24, CSVD *n* = 20). Statistical significance is indicated (*p ≤ 0.05, **p ≤ 0.005, ***p ≤ 0.0005, ****p < 0.0001). Ctrl, control; ALS, amyotrophic lateral sclerosis; Epi, epilepsy; CSVD, cerebral small vessel disease; HA, hypertensive arteriopathy; CAA, cerebral amyloid angiopathy; BCAN, brevican; NCAN, neurocan; CSF, cerebrospinal fluid; cl., cleaved.

**FIGURE 5:**
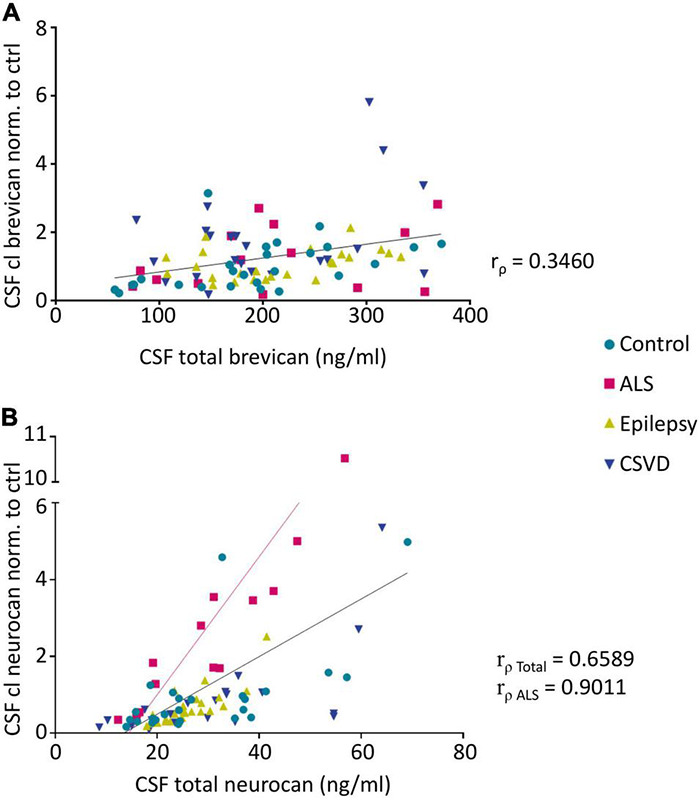
Spearman’s correlation analysis reveals significant correlations between CSF levels of cleaved brevican (normalized to controls) and total brevican (r_ρ_ = 0.346, *p* < 0.05) **(A)** (Pairs: Ctr. *n* = 26, ALS *n* = 14, Epi *n* = 24, CSVD *n* = 22) and also between the CSF levels of cleaved neurocan (normalized to controls) and total neurocan (r_ρ_ = 0.6589, *p* < 0.001) **(B)** (Pairs: Ctr. *n* = 25, ALS *n* = 13, Epi *n* = 24, CSVD *n* = 20) over all groups. Within the ALS group this correlation is strongest (r_ρ_ = 0.9011, *p* < 0.0001). CSF, cerebrospinal fluid, ALS, amyotrophic lateral sclerosis, CSVD, cerebral small vessel disease.

The neo-specific brevican antibody (Valenzuela et al., 2014) detected the human 60kDa brevican fragment in the CSF (see example blot in Figure 4A and in Supplementary Figure 2A). The rank analysis of covariance including the factor group (control, ALS, epilepsy, & CSVD) was neither significant for cleaved brevican (*F*(3,82) = 1.31, *p* = 0.28, η_*p*_^2^ = 0.05) nor for the ratio of cleaved versus total brevican (*F*(3,82) = 1.81, *p* = 0.15, η_*p*_^2^ = 0.06) (Figures 4B,C). Also, for the CSVD subgroups (HA versus CAA) there was no significant difference in cleaved brevican as well as cleaved normalized to total brevican (all *t*s ≤ 1.80, ps ≥ 0.09). Across all groups, cleaved brevican fragment levels significantly correlated with total brevican concentrations (r_ρ_ = 0.3460, *p* < 0.05; Figure 5A).

#### Cleaved Neurocan in the Cerebrospinal Fluid

Human neurocan fragments in the CSF appeared as 150 kDa C-terminal product and approx. 130kDa N-terminal fragment, which is in agreement with previous work (Rauch, 2012; see example blot in Figure 4D and in the Supplementary Figure 2B). The rank analysis of covariance with the factor group (control, ALS, epilepsy, and CSVD) on C-terminal neurocan fragment levels reached statistical significance, *F*(3,78) = 4.35, *p* < 0.01, η_*p*_^2^ = 0.14. As shown in Figure 4E, Tukey’s HSD tests indicated significantly increased mean scores in the ALS versus all other groups (all ps ≤ 0.02). The rank analysis of covariance on cleaved normalized to total neurocan levels revealed a large significant effect of group (control, ALS, epilepsy, and CSVD), *F*(3,78) = 8.48, *p* < 0.001, η_*p*_^2^ = 0.25, with *post hoc* Tukey’s HSD tests again indicating significantly increased mean scores in the ALS compared to all other groups (all ps < 0.001; see Figure 4F). In the ALS group, we also found the descriptively strongest correlation between total neurocan and its cleaved C-terminal fragment (all groups: r_ρ_ = 0.659, *p* < 0.001; ALS: r_ρ_ = 0.90, *p* < 0.001; Figure 5B). No significant differences were found within the CSVD subgroups (CAA versus HA) for cleaved neurocan and cleaved normalized to total neurocan levels (all *t*s ≤ 0.64, ps ≥ 0.53).

### Elucidation of Further Diagnostic Parameters as Confounders

To detect correlations of the estimated CNS-derived proteoglycan concentrations in the patients’ body fluids with each other and with established biomarkers we performed a Spearman’s correlation analysis covering relevant diagnostic parameters obtained in the clinical work up (Figure 3). The serum concentrations of brevican and neurocan did not show any correlations with other parameters.

For the total (r_ρ_ = 0.272, *p* = 0.01) and cleaved brevican levels (r_ρ_ = 0.288, *p* = 0.007) as well as for total neurocan (r_ρ_ = 0.262, *p* = 0.014) in the CSF we detected significant positive correlations with the age of the patients, which did not survive Bonferroni correction. Interestingly, CSF proteoglycan levels, except for cleaved brevican, also correlated strongly with the Alzheimer precursor protein fragment Abeta 1-40, a physiological brain-derived extracellular polypeptide (total neurocan r_ρ_ = 0,747, *p* < 0.001, total brevican r_ρ_ = 0,6, *p* < 0.001, cleaved neurocan r_ρ_ = 0,56, *p* < 0.001). For cleaved as well as for cleaved/total brevican in the CSF, we observed significant correlations with CSF albumin and with the albumin quotient (r_ρ_ = 0.41 and 0.39, respectively, *p* < 0.01).

**FIGURE 6:**
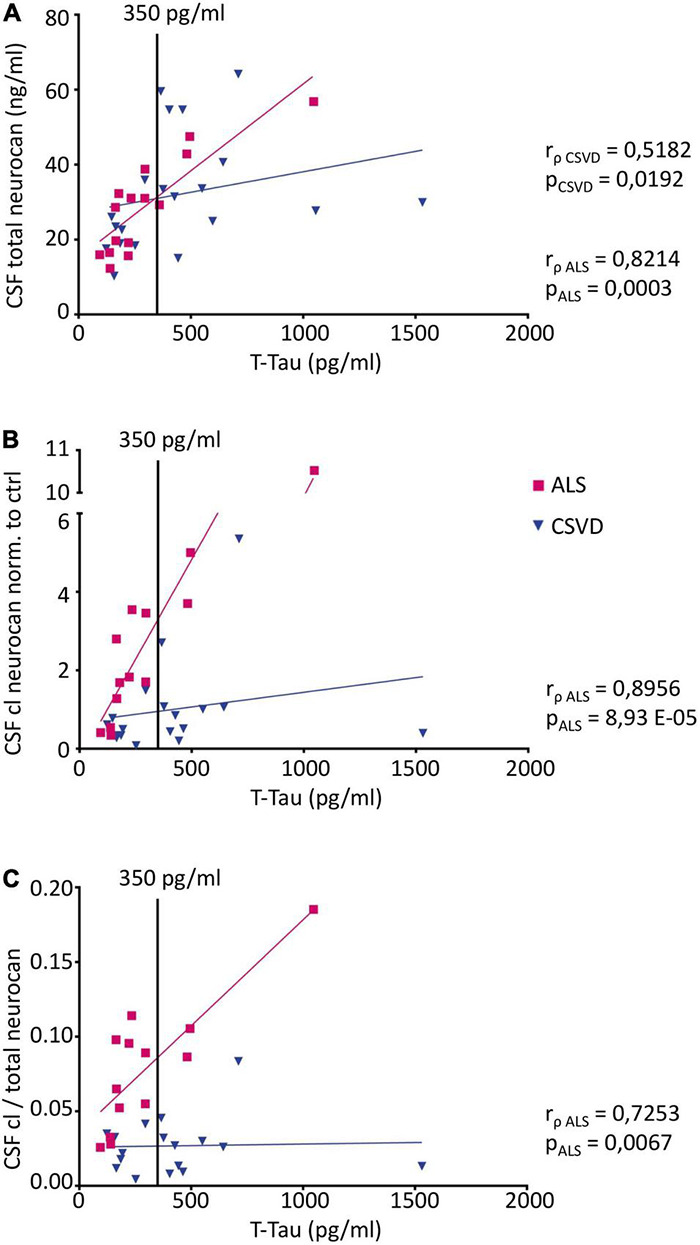
Correlation analysis of CSF neurocan levels with the neurodegeneration marker total Tau (t-Tau) (Pairs CSF NCAN: ALS *n* = 15, CSVD *n* = 20; Pairs CSF cl. NCAN: ALS *n* = 13, CSVD *n* = 18; Pairs CSF cl./total NCAN: ALS *n* = 13, CSVD *n* = 18) **(A)** Total neurocan correlates with t-Tau levels both in the ALS and the CSVD group (ALS: r_ρ_ = 0.8214, *p* = 0.0003; CSVD: r_ρ_ = 0.5182, *p* = 0.0192). For cleaved neurocan **(B)** and for the ratio of cleaved to total neurocan **(C)** correlations with t-Tau were found in the ALS group only (CSF cl. NCAN: r_ρ_ = 0.8956, *p* < 0.0001; CSF cl./total NCAN: r_ρ_ = 0.7253, *p* = 0.0067). The t-Tau threshold value of 350 pg/ml is indicated with a black line.

Since ALS and CSVD are neurodegenerative disorders, we also explored the correlations with neurodegeneration markers NF-L and total Tau. As shown in Figure 6, there were positive correlations with t-Tau for all neurocan measures analyzed in CSF of the ALS samples (in A for total neurocan r_ρ_ = 0.82, *p* < 0.001; in B for cleaved neurocan-C fragment r_ρ_ = 0.90, *p* < 0.001; and in C for the ratio of cleaved to total neurocan r_ρ_ = 0.73, *p* < 0.01). In the CSVD sample, only total neurocan was significantly correlated with t-Tau values (Figure 6A; r_ρ_ = 0.52, *p* < 0.05), and contrary to our expectations we did not find any correlation with NF-L levels in the patients’ CSF samples.

As epilepsy can be caused by neuroinflammatory processes we checked our epilepsy cohort for the occurrence of encephalitis. There were no cases with acute, but 5 cases with putative subacute encephalitis. A comparison between these two subgroups revealed significantly lower amounts of CSF neurocan and neurocan-C in the encephalitis cases (Supplementary Figure 3), however, we did not find a correlation of neurocan levels with the IgG index across groups (Figure 3).

## Discussion

Here we show in a cohort of neurological patients that the CSF levels of the major proteolytic fragment of the perineuronal ECM proteoglycan neurocan, neurocan-C, are significantly higher in the ALS group as compared to controls, but also to epilepsy and CSVD patients. This effect became even more evident after normalizing concentrations of the cleaved to total core proteoglycan. The lack of significant findings from serum samples may be due to the more distal relation of serum to brain processes, but mostly due to the large dynamic range of plasma proteins and the huge intra- and inter-individual variability in serum protein composition. The low abundance of neurocan is another factor contributing to the lack of significant findings in serum samples.

### Proteoglycan Fragments as Readout for Proteolytic Activity

The neural ECM has a relatively low turnover rate (i.e., within days; see Fawcett et al. (2019)), with its biosynthesis and degradation being in equilibrium under healthy conditions keeping homeostasis (Dityatev et al., 2010). Nevertheless, the composition and integrity of the brain ECM can be dynamically adapted to the activity state of neural networks (i.e., within minutes to hours), mostly *via* the action of ECM-cleaving matrix metalloproteinases (Frischknecht et al., 2014). Thus, strictly controlled proteolytic events yield a more loosened ECM which allows structural changes in the synaptic wiring and functional connectivity alterations. Importantly, dysregulation of these ECM-shaping processes can be a hallmark for several neurological disorders (Gottschall and Howell, 2015), making glycoprotein or proteoglycan fragments in body fluids of patients valuable readouts for the general ECM proteolytic activity. This activity may vary largely with disease stages and severity and may therefore be an additional proxy for the disease progression. For neurocan it was shown that the major proteolytic event yielding the neurocan-C fragment is affected by cytokines like Transforming Growth Factor beta (TGFbeta), Platelet-Derived Growth Factor (PDGF), and Epidermal Growth Factor (EGF) (Asher et al., 2000).

Furthermore, next to the proteolytic activity by itself (both in the brain parenchyma and in the CSF) also the passing rate across the BBB resp. the blood CSF barrier may affect the levels measured in body fluids. This assumption is supported by the observed correlation of cleaved versus total brevican in the CSF with the albumin quotient (Figure 3), arguing for increased barrier leakage driving the occurrence of the proteoglycan fragment (which is in the same size range as albumin with 66 kDa) in the CSF.

Finally, the glymphatic flux rate inside the brain parenchyma is supposedly a major factor in the regulation of the CSF composition. This assumption is supported by our finding that CSF proteoglycan levels largely correlate with the levels of Abeta 1-40, another neural polypeptide found in the extracellular space of the CNS and cleared by the glymphatic system (Iliff et al., 2012), indicating common efflux mechanisms to the CSF. However, cleaved neurocan shows also an effect of the disease group (ALS), pointing to a disease-specific mechanism not explained by physiological clearance rates.

### Links to Neurological Disorders

The neural ECM is a dynamic molecular filter for all cellular secretomes in the brain. It undergoes constant homeostatic remodeling and appears disorganized in several pathologies. So far, to our knowledge no other study investigated brevican and neurocan fragments as potential biomarkers for epilepsy, CSVD or ALS. However, in a series of biochemical or proteomic studies a Swedish group investigated brevican and neurocan already in a spectrum of other neurological conditions. In 2018 they demonstrated both brevican and neurocan CSF levels being lower after prophylactic cerebral radiotherapy of lung cancer patients, but only in cases without brain metastases (Fernström et al., 2018). In TBI patients the same group showed that CSF levels of brevican and neurocan as well as of their binding partners tenascin-C and tenascin-R change over time after the injury – again arguing for a disease stage dependence of ECM modifications - and that lower values correlated with a favorable outcome for the TBI patients (Minta et al., 2019a). For neurocan they found a reduction in the serum of TBI cases. In a follow-up study, they demonstrated that mostly N-terminal brevican peptides (present on the 60 kDa fragment investigated here) were reduced in TBI as compared to idiopathic normal pressure hydrocephalus (iNPH) CSF samples as contrast group, and that ADAMTS-like proteolytic activity in the CSF of TBI patients was increased (Minta et al., 2021b). Interestingly, in their study the rise in ADAMTS-like activity correlated also with matrix metalloprotease (MMPs 1, 2, 3, 10) concentrations in CSF, suggesting a general increase in extracellular proteolytic activity. In iNPH patients the same group compared lumbar and ventricular CSF concentrations of proteoglycans and MMPs demonstrating that neither brevican nor neurocan differed between CSF compartments, however, MMPs −1, −2, −10 and the tissue inhibitor of metalloproteases TIMP-1 were found increased in lumbar as compared to ventricular CSF (Minta et al., 2021c).

For different types of dementias specific correlations with neural proteoglycans have been reported: In AD patients the CSF and serum levels of brevican or neurocan were concurringly found to be unchanged as compared to non-demented controls and there was no correlation with the Abeta_42/40_ values but a significant correlation between brevican, neurocan, and the brevican-interacting ECM glycoprotein tenascin-R (rho = 0.68–0.77, *p* < 0.05) (Begcevic et al., 2018; Brinkmalm et al., 2018; Minta et al., 2019b,2021a; Jonesco et al., 2020).

#### Vascular Dementia and Cerebral Small Vessel Disease

With the help of a fragment-specific anti-brevican antibody, Jonesco and colleagues found in the serum of a highly heterogeneous group of patients with other dementias (including mostly vascular and fronto-temporal lobar dementia, but also Lewy body dementia, Parkinson’s disease, normal pressure hydrocephalus, depressive pseudo-dementia, apoplexia, aphasia, paraneoplasia, epilepsy, hippocampal atrophy, and pick’s disease cases) slightly reduced amounts of total brevican but increased levels of the C-terminal brevican fragment derived from ADAMTS-4 cleavage, yielding a clearly increased ratio of cleaved over total brevican (Jonesco et al., 2020). In our clinically more homogeneous CSVD cohort we did not find any differences to the other groups (Figure 4B) and also not between the two subgroups HA and CAA. In contrast to Jonesco et al. and to our data, Minta and coworkers tested for the abundance of neurocan- and brevican-derived peptides in the CSF of two small cohorts of vascular dementia patients and found in the 1st cohort all peptides significantly reduced as compared to control subjects and AD patients, and in their 2nd cohort two N-terminal brevican peptides remained significantly diminished (Minta et al., 2021a), demonstrating the need for detailed analysis of demographic as well as disease-specific confounding factors in the cohorts.

#### Amyotrophic Lateral Sclerosis

This motoneuron disease is considered as non-cell autonomous, i.e., beyond the motor neurons also the surrounding glial cells are affected (Galbiati et al., 2020). To come closer to the mechanisms behind elevated levels of neurocan-C in our ALS group we looked for correlations with clinical measures like ALS diagnosis according to El Escorial criteria, disease severity (as expressed by ALSFRS-R score and ECAS score, placement of a percutaneous endoscopic gastrostomy (PEG) or non-invasive ventilation (NIV)) and disease duration in our sample. However, we did not find any significant correlations with cleaved neurocan levels, probably because our sample size is actually too low to form instructive subgroups. Data from a larger cohort need to be collected which will allow a reliable stratification of ALS patients according to El Escorial criteria.

Interestingly, in ALS conditions two of the factors known to stimulate neurocan cleavage, namely EGF and TGFbeta (Asher et al., 2000) are affected: at later stages of ALS, the TGFbeta pathway is persistently upregulated, leading, e.g., to excessive activation of microglia (Galbiati et al., 2020), and inhibitors of the EGF receptor have proven to be beneficial in the SOD1 mouse model of ALS (Le Pichon et al., 2013). These findings make it tempting to speculate that dysregulated cytokine levels may cause the observed effect, a hypothesis which could be tested in larger patient groups.

A proteomic study in ALS patients showed no differences for brevican but a significant downregulation of total neurocan (*p* = 0,0067; −0,39-fold change) in the CSF samples as compared to healthy control samples (Bereman et al., 2018). The difference to our findings may be due to sample size differences (33 ALS patients in Bereman et al. (2018) versus 15 cases in our sample), age differences (40-55 yrs. versus 64 yrs.) or to the different methodology (proteomics versus immunoblot).

#### Epilepsy

A study in mesial temporal lobe epilepsy patients (Perosa et al., 2002) showed upregulated chondroitin sulfates and hyaluronic acid in the post mortem hippocampus, but no differences in the CSF. Conflicting data have been published for total brevican levels in epilepsy. While one study found *via* immunoblot of post mortem human epilepsy hippocampus samples a reduction of total brevican immunoreactivity (Favuzzi et al., 2017) a more recent study reported upregulated brevican levels in human post mortem frontal cortex samples from epilepsy patients as compared to controls (Pires et al., 2021).

Inflammatory mechanisms, which are quite common in epilepsy cases, may severely affect both ECM cleavage and clearance from brain tissue. In our sample we had no cases with acute inflammation, but a few cases with putative subacute encephalitis. Indeed, in these cases CSF neurocan levels were significantly lower than in patients without inflammation, but we did not find a correlation with the IgG index as proxy for intrathecal immunoglobulin synthesis. Only CSF brevican levels correlate with the IgG index, suggesting that there may be a disease-independent effect of neuroinflammation is of potential diagnostic and also mechanistic interest. However, it is early times to formulate a specific hypothesis about the particular role of neuroinflammatory mechanisms and the specific occurrence of proteoglycan fragments in the CSF, and further studies with larger case numbers with specified epilepsy syndromes are needed.

### Proteoglycan Fragments as Matrikines

Fragments produced by proteolytic cleavage of CNS proteoglycans may not only be an indicator for the deconstruction of the intact ECM, but they could provide a gain of function by acting as matrikines. This term describes bioactive proteolytic (or glycosidic) fragments exerting signaling functions which may be different from the role of the uncleaved ECM components (Ricard-Blum and Salza, 2014; Fontanil et al., 2021).

Although transport modalities for brain-derived matrikines into the CSF and into the serum are still fully unclear, to study them under pathophysiological conditions could be of interest both from a clinical but also from a cell biological perspective.

### Limitations of This Study and Outlook

Further experiments are necessary to confirm these findings. in an independent sample and to clarify the clinical value.

The statistical power from the relatively small numbers of individuals involved was felt to be too low to draw firm conclusions with regard to the suitability and disease specificity as bona fide biomarkers, however, our results are promising and deserve further validation in larger cohorts to unravel the full potential in the clinical work up, not least to come closer to the pathophysiological mechanisms behind our observations.

Taken together, our experiments suggest that – beyond association with clinical phenotypes – the CSF levels of the neural proteoglycans investigated here seem to be differentially regulated: while total brevican and neurocan as well as the C-terminal fragment of neurocan appear to be largely controlled by the general glymphatic flux, the 60kDa brevican fragment seems to be rather controlled by the BBB function.

From our work we conclude that proteolytic cleavage products of brain-derived perineuronal ECM molecules, such as neurocan fragments, may allow insights into the integrity of the brain’s extracellular environment, being potent CSF indicators for the actual proteolytic ECM fragmentation activity in the CNS tissue, especially if normalized to the concentration of total core proteoglycans. In the clinic, their correlation with established markers for neurodegeneration or BBB leakage may pave the way to a more differentiated diagnosis of neurological disorders, based on body fluids. Our results suggest to include the ratio of cleaved to total neurocan into informative biomarker panels to improve the discriminatory power between disorders.

## Data Availability Statement

The original contributions presented in the study are included in the article/Supplementary Material, further inquiries can be directed to the corresponding author/s.

## Ethics Statement

The studies involving human participants were reviewed and approved by Ethics Committee of the Otto von Guericke University Magdeburg. The patients/participants provided their written informed consent to participate in this study.

## Author Contributions

CIS, EG and SS conceptualized the study. SS, CG, FS and SV assessed the clinical phenotypes and provided patient samples. CG provided the clinical data. WH and LH performed the experiments and designed the figures. CS and WH performed data analysis. CIS and EG designed the experiments and wrote the draft. All authors revised and finalized the manuscript and approved the submitted version.

## Conflict of Interest

The authors declare that the research was conducted in the absence of any commercial or financial relationships that could be construed as a potential conflict of interest.

## Publisher’s Note

All claims expressed in this article are solely those of the authors and do not necessarily represent those of their affiliated organizations, or those of the publisher, the editors and the reviewers. Any product that may be evaluated in this article, or claim that may be made by its manufacturer, is not guaranteed or endorsed by the publisher.

## Supplementary Material

Supplementary Figure 1 The neo antibody recognizes the human brevican fragment in the CSF.Western blot showing the result of the antigen competition experiment to prove specificity of theantibody directed against the C-terminal neoepitope of the N-terminal brevican fragment. In humanCSF the antibody shows reactivity with a 60kDa protein band (lane A, arrow) which is clearlyreduced after preincubating the antibody with a 100fold molar excess of the rat brevican peptide usedfor immunization (lane D). The band at 70kDa results from cross-reactivity with albumin (asterisk),which is not affected by the competition experiment. In soluble fractions of human temporal (tCtx;B) and frontal (fCtx; C) cortex the antibody does not show any blockable specificity in the 60kDarange (lanes E, F). Protein marker (M) sizes are indicated (in kDa).

Supplementary Figure 2 Example immunoblots of cleaved brevican **(A)** and neurocan **(B)** immunoreactivity in individual CSF samples and a reference sample loaded on all gels forcalibration. Protein marker (M) sizes are indicated (in kDa). Ctrl – control; HA – hypertensivearteriopathy; CAA – cerebral amyloid angiopathy; ALS – amyotrophic lateral sclerosis; Ref –reference sample. Quantified bands are indicated with arrows. Asterisk marks the nonspecificallylabelled albumin band.

Supplementary Figure 3 Neurocan levels in the CSF of epilepsy patients with or without subacuteencephalitis. Group-wise comparison of z-scores from age- and sex-corrected total concentrations ofneurocan as measured *via* ELISA **(A)**, and of the cleaved neurocan-C fragment as measured *via*immunoblot **(B)**. Statistical significance is indicated.
