# Supplementary figures and images for: Brevican and Neurocan Cleavage Products in the Cerebrospinal Fluid - Differential Occurrence in ALS, Epilepsy and Small Vessel Disease
Source: Front Cell Neurosci. 2022 Apr 11;16:838432. doi: 10.3389/fncel.2022.838432 (PMC9036369; doi:10.3389/fncel.2022.838432)

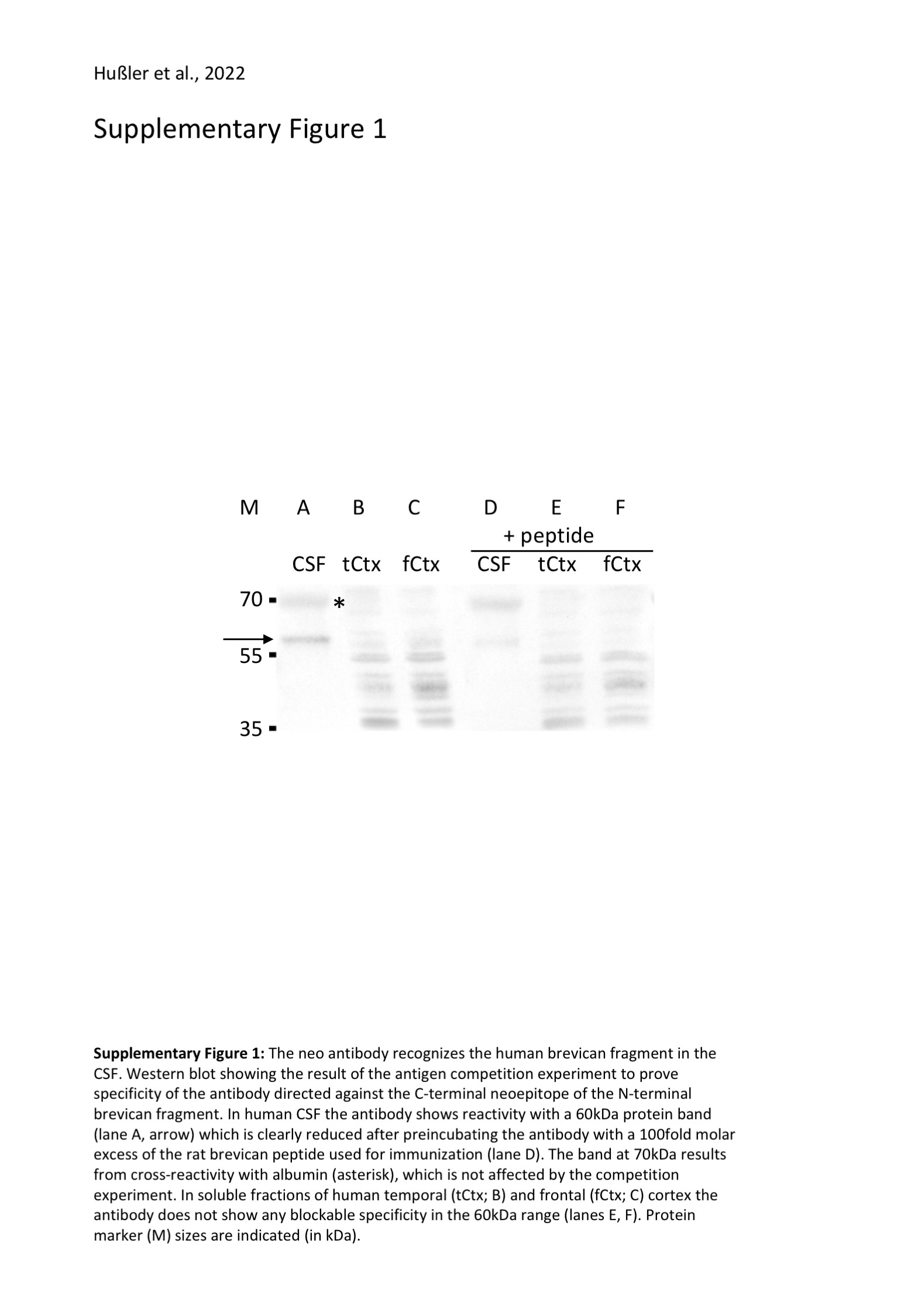

Supplement: Supplementary Figure 1 — The neo antibody recognizes the human brevican fragment in the CSF.Western blot showing the result of the antigen competition experiment to prove specificity of theantibody directed against the C-terminal neoepitope of the N-terminal brevican fragment. In humanCSF the antibody shows reactivity with a 60kDa protein band (lane A, arrow) which is clearlyreduced after preincubating the antibody with a 100fold molar excess of the rat brevican peptide usedfor immunization (lane D). The band at 70kDa results from cross-reactivity with albumin (asterisk),which is not affected by the competition experiment. In soluble fractions of human temporal (tCtx;B) and frontal (fCtx; C) cortex the antibody does not show any blockable specificity in the 60kDarange (lanes E, F). Protein marker (M) sizes are indicated (in kDa). [file Image_1.jpg]

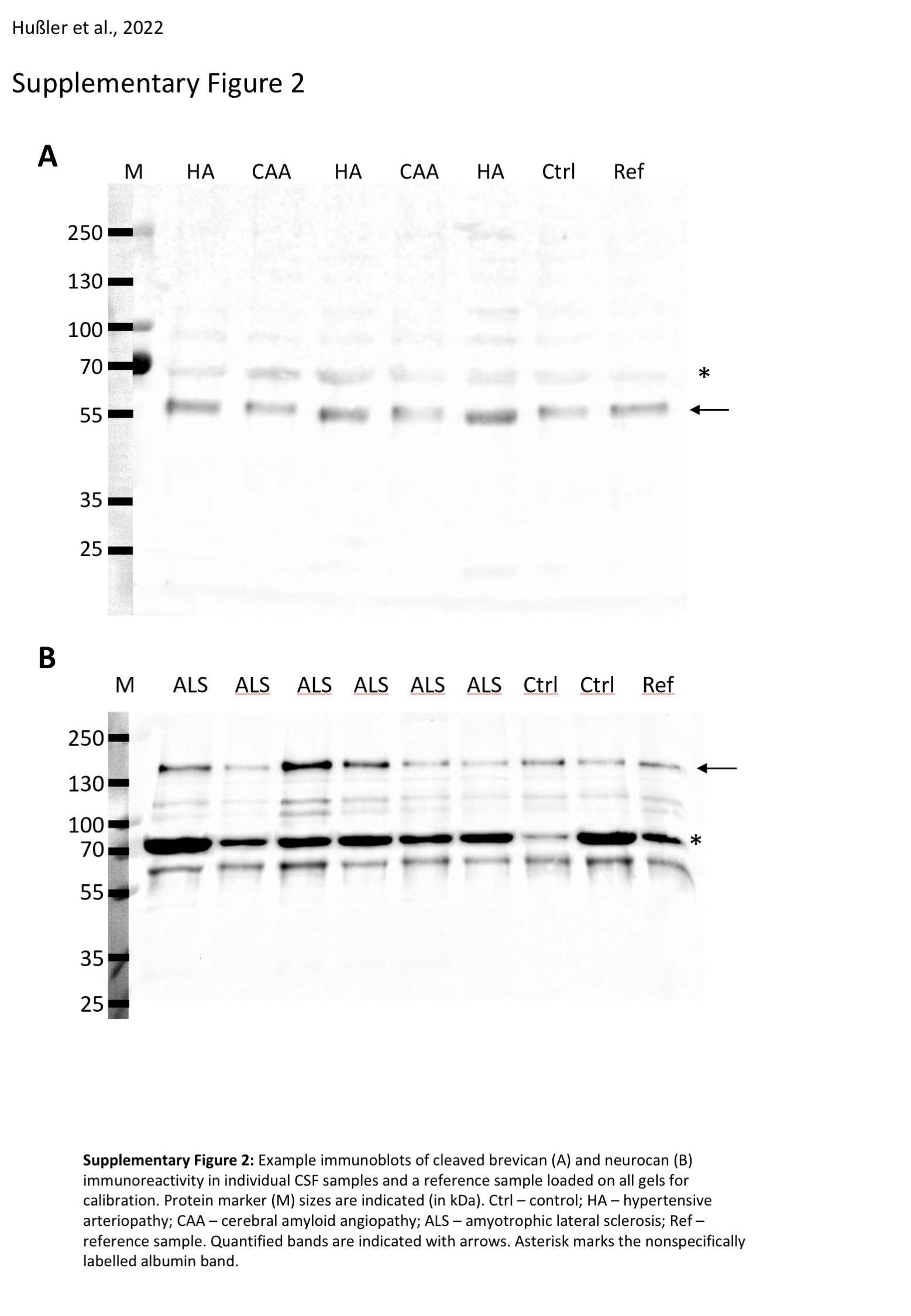

Supplement: Supplementary Figure 2 — Example immunoblots of cleaved brevican (A) and neurocan (B) immunoreactivity in individual CSF samples and a reference sample loaded on all gels forcalibration. Protein marker (M) sizes are indicated (in kDa). Ctrl – control; HA – hypertensivearteriopathy; CAA – cerebral amyloid angiopathy; ALS – amyotrophic lateral sclerosis; Ref –reference sample. Quantified bands are indicated with arrows. Asterisk marks the nonspecificallylabelled albumin band. [file Image_2.jpg]

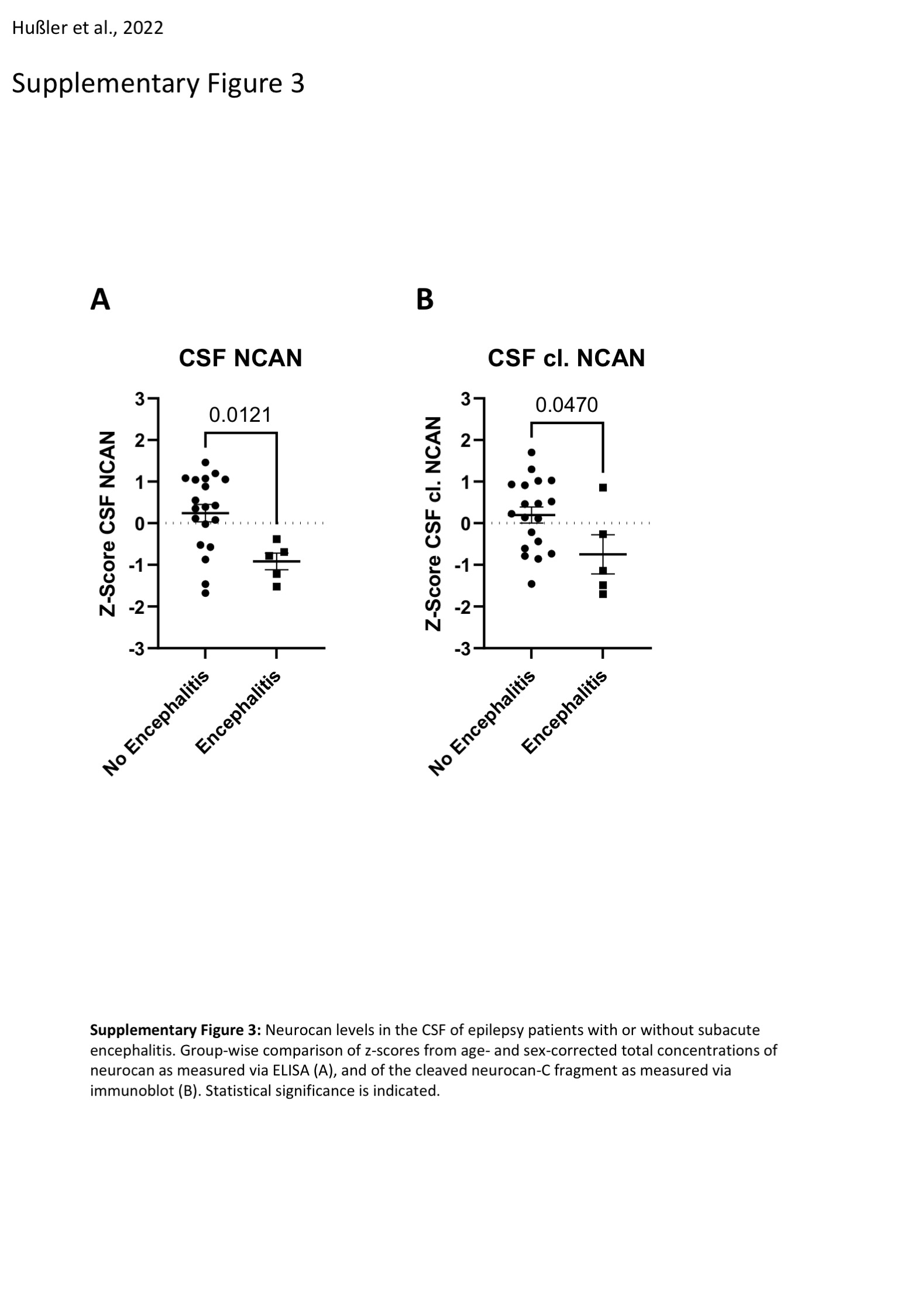

Supplement: Supplementary Figure 3 — Neurocan levels in the CSF of epilepsy patients with or without subacuteencephalitis. Group-wise comparison of z-scores from age- and sex-corrected total concentrations ofneurocan as measured via ELISA (A), and of the cleaved neurocan-C fragment as measured viaimmunoblot (B). Statistical significance is indicated. [file Image_3.jpg]
