# Supplementary material for: Brevican and Neurocan Cleavage Products in the Cerebrospinal Fluid - Differential Occurrence in ALS, Epilepsy and Small Vessel Disease
Source: Front Cell Neurosci. 2022 Apr 11;16:838432. doi: 10.3389/fncel.2022.838432 (PMC9036369; doi:10.3389/fncel.2022.838432)
Supplement: Supplementary file 4 [file Data_Sheet_1.docx]

**Hußler et al., 2022**

**Supplementary Tables 1 and 2: Kruskal-Wallis one-way analyses on uncorrected data**

| **Supplementary Table S1. Kruskal-Wallis tests of the factor group (control, ALS, epilepsy, & CSVD) on uncorrected brevican and neurocan data** | | | |
| --- | --- | --- | --- |
| Dependent variable | χ^2^ | *p* | η^2^ |
| CSF BCAN | 0.77 | 0.86 | 0.01 |
| CSF NCAN | 0.75 | 0.86 | 0.01 |
| Serum BCAN | 2.02 | 0.57 | 0.03 |
| Serum NCAN | 6.66 | 0.08 | 0.08 |
| cl. BCAN | 6.89 | 0.08 | 0.08 |
| cl. NCAN | 12.84 | < 0.01 | 0.16 |
| cl. BCAN/total BCAN | 8.73 | 0.03 | 0.10 |
| cl. NCAN/total NCAN | 21.20 | < 0.001 | 0.26 |

While there was a significant group effect of cleaved brevican (cl. BCAN) normalized to the total BCAN levels, none of the post-hoc comparisons reached statistical significance (all adjusted *p*s ≥ 0.05; control: M = 37.85; ALS = 46.50; epilepsy: M = 36.58; CSVD: M = 55.82).

The significant group effect on cl. NCAN goes back to significant differences (all adjusted ps < 0.05) between the ALS group (M = 62.92) and the other groups (control: M = 39.60; epilepsy: M = 36.75.00; CSVD: M = 35.65). For cl. NCAN normalized to the total NCAN levels there was a similar result pattern with the ALS group showing increased mean rank scores (ALS: M = 69.23) compared to the other groups (control: M = 38.00; epilepsy: M = 36.00; CSVD: M = 34.45), all adjusted *p*s < 0.001.

| **Supplementary Table S2. Results of the within CSVD group comparisons test (CAA vs. HA) with the Mann-Whitney-U test** | | | |
| --- | --- | --- | --- |
| Dependent variable | *U* | *p* | η^2^ |
| CSF BCAN | 76.00 | 0.33 | 0.05 |
| CSF NCAN | 66.00 | 0.75 | 0.01 |
| Serum BCAN | 61.00 | 0.37 | 0.39 |
| Serum NCAN | 0.39 | 0.95 | <0.01 |
| cl. BCAN | 85.00 | 0.12 | 0.12 |
| cl. NCAN | 40.00 | 0.50 | 0.03 |
| cl. BCAN/total BCAN | 77.00 | 0.30 | 0.06 |
| cl. NCAN/total NCAN | 43.00 | 0.66 | 0.01 |
